# First report of *Cryptosporidium* spp. in white yaks in China

**DOI:** 10.1186/1756-3305-7-230

**Published:** 2014-05-19

**Authors:** Si-Yuan Qin, Xiao-Xuan Zhang, Guang-Hui Zhao, Dong-Hui Zhou, Ming-Yang Yin, Quan Zhao, Xing-Quan Zhu

**Affiliations:** 1 State Key Laboratory of Veterinary Etiological Biology, Key Laboratory of Veterinary Parasitology of Gansu Province, Lanzhou Veterinary Research Institute, Chinese Academy of Agricultural Sciences, Lanzhou, Gansu Province 730046, PR China; 2 College of Animal Science and Technology, Jilin Agricultural University, Changchun, Jilin Province 130118, PR China; 3 College of Veterinary Medicine, Northwest A&F University, Yangling, Shaanxi Province, 712100, PR China

**Keywords:** *Cryptosporidium* spp, Genetic characterization, Prevalence, White Yak, China

## Abstract

**Background:**

*Cryptosporidium* is an enteric apicomplexan parasite, which can infect yaks, leading to reduction of milk production and poor weight gain. White yak (*Bos grunniens*) is a unique yak breed inhabiting only in Tianzhu Tibetan Autonomous County, Gansu province, northwestern China. The objective of the present study was to molecularly determine *Cryptosporidium* infection and species in white yaks.

**Findings:**

Seventy-six fecal samples from white yaks in Tianzhu Tibetan Autonomous County, Gansu province were collected. The small subunit ribosomal RNA (SSU rRNA) gene of each sample was amplified using nested PCR and sequenced. The *Cryptosporidium* species was determined by comparison of the obtained sequences with that of corresponding *Cryptosporidium* sequences available in GenBank by BLAST (http://www.ncbi.nlm.nih.gov/BLAST/) and phylogenetic analysis with maximum likelihood (ML) using PAUP^*^. The overall prevalence of *Cryptosporidium* infection in white yak was 5.26% (4/76). Species identification showed *C. andersoni* in one sample (collected in September), and *C. bovis* in three samples (one collected in November and two collected in September).

**Conclusions:**

The present investigation revealed the existence of *Cryptosporidium* infection in white yaks in China, for the first time, and two *Cryptosporidium* species, namely *C. andersoni* and *C. bovis*, were identified. These findings extend the host range for *Cryptosporidium* spp., and also provide base-line information for further studies of molecular epidemiology and control of *Cryptosporidium* infection in the unique white yaks.

## Findings

### Background

*Cryptosporidium* is an apicomplexan, enteric parasite pathogen, which may lead to diarrheal illness and other severe diseases of animals and humans [1,2]. Human infection with *Cryptosporidium* is usually through close contact with infected animals or consuming contaminated water or food [3]. Many animals can be infected with *Cryptosporidium* spp. including livestock and wild animals [4-8]. Four *Cryptosporidium* species have been identified in black yaks in previous studies [5,7].

White yak (*Bos grunniens*) is a unique yak breed living only in Tianzhu Tibetan Autonomous County, Gansu province, northwestern China, where the air pressure is high and the temperature and oxygen content are low. White yak is known as the pearl of the plateau, and it is a rare and precious semi-wild animal of China and the world. Only approximately 49,400 white yaks were available in Tianzhu Tibetan Autonomous County [9]. Milk and meat of white yaks are the sought-after delicacy for local Tibetan people and other residents in Gansu Province. Previous studies have indicated that *Cryptosporidium* could infect black yaks, but it is yet to know whether white yaks are infected with *Cryptosporidium* spp.. The objective of the present study was to determine *Cryptosporidium* infection and species in white yaks in Tianzhu Tibetan Autonomous County, Gansu Province, China.

## Methods

### Ethics statement

This study was approved by the Animal Ethics Committee of Lanzhou Veterinary Research Institute, Chinese Academy of Agricultural Sciences (Approval No. LVRIAEC2013-010). The white yaks from which the feces were collected, were handled in accordance with good animal practices required by the Animal Ethics Procedures and Guidelines of the People’s Republic of China.

### The study site

The fecal samples used in the present study were collected from white yaks in Tianzhu Tibetan Autonomous County, Gansu Province, northwestern China. The sampling site is between the eastern longitudes of 102°07′-103°46′ and northern latitudes of 36°31′-37°55′. The altitude of Tianzhu Tibetan Autonomous County ranges from 2,040 m to 4,878 m, and the average annual temperature are from -8°C to 4°C, respectively.

### Specimen collection

A total of 76 white yak fecal samples were randomly collected from two farms between September and November 2013 in Tianzhu Tibetan Autonomous County, Gansu Province, northwestern China. Information about the examined white yaks was obtained from the herdsmen at the time of sample collection. Fecal samples were collected with sterile gloves and transported to the laboratory in Lanzhou Veterinary Research Institute, Chinese Academy of Agricultural Sciences, Lanzhou, Gansu Province, China, and stored at 4°C within one week before being tested.

### DNA extraction and PCR amplification

Genomic DNA was extracted from each fecal sample using the Stool DNA kit (OMEGA, USA) according to the manufacturer’s instructions, and stored at -20°C until PCR amplification. The small subunit ribosomal RNA (SSU rRNA) gene was amplified using nested PCR to determine species/genotypes of *Cryptosporidium* spp. [4]. Positive and negative controls were included in each amplification. Amplification products were examined using 1.5% agarose gel containing GoldView (Solarbio, China) and were observed under UV light.

### Sequencing and phylogenetic analyses

All positive secondary PCR products were directly sequenced by Sangon Biotech Company (Shanghai, China). The obtained sequences were aligned with *Cryptosporidium* sequences available in GenBank database using the BLAST (http://www.ncbi.nlm.nih.gov/BLAST/) and Multiple Sequence Alignment Program, Clustal X 1.83 [4]. Phylogenetic relationships of *Cryptosporidium* spp. were re-constructed using Maximum Likelihood (ML) method, which were performed using a Fitch criterion within PAUP^*^ (version 4.0b4a) and 1000 non-parametric bootstrap replicates. *Plasmodium cathemerium* (GenBank accession number: AY625607) was used as the out-group. The sequences obtained in the present study were deposited in GenBank with accession numbers of KJ094571 and KJ094572.

## Results and discussion

**Figure 1:**
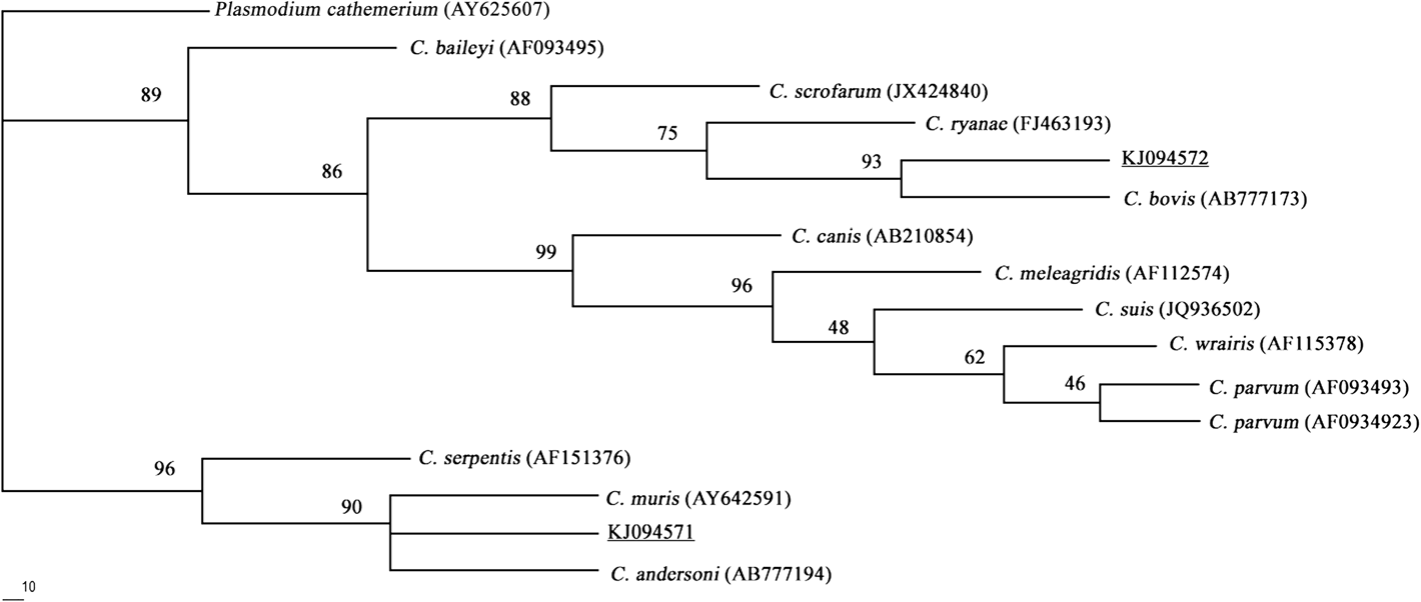
**Phylogenetic analyses of *Cryptosporidium *spp. using Maximum Likelihood (ML) method based on sequences of the small subunit ribosomal RNA (SSU rRNA) gene.** The numbers at clades indicate bootstrap values. The *Cryptosporidium* isolates identified in the present study are underlined.

Of 76 examined fecal samples of white yaks collected in Tianzhu Tibetan Autonomous County, Gansu province, 4 (5.26%) were positive for *Cryptosporidium* infection. Sequencing and phylogenetic analysis identified two *Cryptosporidium* species present in these samples (Figure 1). One *C. andersoni*- and two *C. bovis*-positive samples were detected in September, and one *C. bovis*-positive sample was collected in November.

The overall *Cryptosporidium* prevalence in Tianzhu white yaks was 5.26% (4/76) using a molecular approach, which was lower than that in yaks investigated by microscopy (10.4%) [10], serological test (33.64%) [11] and PCR (24.2%) [5] in Shanghai city and Qinghai province of China. The lower prevalence of *Cryptosporidium* infection in Tianzhu white yaks determined in the present investigation is likely due to the cold sampling seasons (September to November are becoming cold in Tianzhu Tibetan Autonomous County) and the small sample size.

Five *Cryptosporidium* species/genotypes, namely *C. parvum*[5], *C. ryanae*[5], *Cryptosporidium* sp. z13 [7], *C. bovis*[5] and deer-like genotype [12], have been detected in yaks in China. The present study revealed the presence of *C. andersoni* and *C. bovis* in white yaks. Of the four *Cryptosporidium*-positive samples, three represented *C. bovis,* indicating that *C. bovis* is the more prevalent *Cryptosporidium* species in white yaks, which is similar to previous studies that *C. bovis* is more prevalent in water buffaloes and beef calves [13,14].

However, *C. parvum*[5], *C. ryanae*[5], *Cryptosporidium* sp. z13 [7] and deer-like genotype [12] were not detected in white yaks in the present study, which may due to the small number of samples examined. *C. parvum* is one of the most important *Cryptosporidium* species, which has public health concerns [15], and it is a common species found in pre-weaned cattle in China and other countries [16-19]. Further studies will sample more white yaks in different seasons of the year to determine the dynamics and full profiles of *Cryptosporidium* infection in white yaks, to examine the infection status of the local Tibetans with *Cryptosporidium*, and to assess the zoonotic potential of *Cryptosporidium* from white yaks.

## Conclusions

The present investigation revealed the existence of *C. andersoni* and *C. bovis* in Tianzhu white yaks, which is the first report of *Cryptosporidium* infection in Tianzhu white yaks. These results not only extend the host range for *Cryptosporidium* spp., but also provide base-line information for further studies of the molecular epidemiology and control of *Cryptosporidium* infection in Tianzhu white yaks.

## Competing interests

The authors declare that they have no competing interests.

## Authors’ contributions

XQZ conceived and designed the study, and critically revised the manuscript. SYQ, XXZ, GHZ and DHZ performed the experiments, analyzed the data and drafted the manuscript. MYY and QZ helped in study design, study implementation and manuscript revision. All authors read and approved the final manuscript.
